# Crystal Chemistry of a Biodegradable Mg_65_Ca_28_Au_7_ Alloy: Thermal Evolution and Refinement of CaAuMg_2_

**DOI:** 10.3390/ma19214654

**Published:** 2026-10-31

**Authors:** Karel Saksl, Zuzana Molčanová, Beáta Ballóková, Renáta Chromá

**Affiliations:** 1 Faculty of Mechanical Engineering, Technical University of Košice, Park Komenského 11, 042 00 Košice, Slovakia; renata.chroma@tuke.sk; 2 Institute of Materials Research of Slovak Academy of Sciences, Watsonova 47, 040 01 Košice, Slovakia; molcanova@saske.sk (Z.M.); bballokova@saske.sk (B.B.)

**Keywords:** Mg–Ca–Au, phase evolution, CaAuMg_2_, Rietveld refinement, rapid solidification, biodegradable alloys

## Abstract

Phase constitution affects localized dissolution and mechanical retention in biodegradable magnesium alloys. We examined melt-spun nominal Mg_65_Ca_28_Au_7_ by X-ray diffraction before and after annealing at differential scanning calorimetry (DSC). Area EDX estimated 64.4 at.% Mg, 26.9 at.% Ca and 8.7 at.% Au. The as-spun pattern is described by Mg_2_Ca and CaAuMg_2_ with normalized crystalline fractions of 56.9 and 43.1 wt.%. After-DSC heating in argon to 400 °C, Mg_3_Au is clearly resolved alongside both phases, with fractions of 51.8 wt.% Mg_2_Ca, 37.8 wt.% CaAuMg_2_ and 10.5 wt.% Mg_3_Au. Mg_3_Au is not securely distinguishable before heating; its absence is not established. Rietveld refinements yield *R*_wp_ = 2.06% and 3.54%. The after-DSC pattern permits refinement of four CaAuMg_2_ positional parameters in Cmcm, with a = 4.3121(1), b = 10.7642(2) and c = 8.2596(2) Å. Au-centered Mg_6_ prisms retain the known MgCuAl_2_ topology; detailed distortions remain texture-sensitive. No significant localized DSC transition is resolved. Phase fractions remain semiquantitative. The results provide a structural basis for subsequent degradation studies; improved corrosion resistance or biological performance is not demonstrated.

## 1. Introduction

Biodegradable magnesium alloys are investigated for temporary internal implants that support tissue during healing and subsequently lose their mechanical function through controlled degradation. Their appeal is the possibility of reducing the need for implant removal while avoiding a permanently retained fixation device. The essential materials problem is to reconcile degradation with the required period of mechanical support. Experiments on Mg alloys show composition-dependent corrosion and bone responses, while work on binary Mg–Ca links increasing Mg_2_Ca content to faster degradation [1,2]. A clinical pilot study of MgYREZr fixation screws illustrates the feasibility of the broader approach in selected orthopedic applications [3]. These results motivate alloy development but do not establish the suitability of the composition studied here.

For a biodegradable alloy, the relevant structural variables include phase identity, elemental partitioning, phase distribution, and the connectivity of interfaces. When constituents differ in electrochemical response, their coupling can promote localized dissolution and change the rate at which load-bearing material is lost. Consequently, the same nominal composition can behave differently after rapid solidification and thermal treatment. The recent review by Song et al. discusses the interplay of alloying, surface modification, degradation, and antibacterial performance in Mg-based biomaterials [4].

A direct demonstration of the phase–degradation relationship was provided by Jia et al.: high-pressure solution treatment suppressed the Mg_2_Ca second phase in a dilute Mg–Ca alloy and produced a more uniform corrosion mode with improved corrosion resistance [5]. This establishes the relevance of phase constitution, but the result cannot be transferred quantitatively to the present Ca-rich, intermetallic-containing ribbon. In particular, identifying a crystal structure does not determine its corrosion potential or dissolution kinetics. Phase-resolved structural characterization is therefore a necessary starting point for testing how processing modifies degradation, rather than a substitute for corrosion and biological measurements.

Gold addition offers a further design opportunity: increased X-ray attenuation may make an implant easier to locate and monitor using computed tomography (CT). The high atomic number of Au and its strong attenuation at diagnostic X-ray energies provide the physical basis; experiments with gold contrast agents demonstrate this radiographic potential [6,7]. Incorporating Au as a minor atomic constituent of a Mg-based alloy is therefore attractive for imaging. Actual CT visibility depends on Au content and distribution, implant dimensions, and scanning conditions. It has not been measured for the present ribbon, and the fate of Au-containing residues during degradation remains to be established.

The Mg–Ca–Au series was introduced by Molčanová et al. as a source of candidate biodegradable materials [8]. For the crystalline alloy reported as Mg_64_Ca_27_Au_9_, corresponding to the rounded EDX designation used for the present material, that study measured an indentation modulus *E*_IT_ = 34 ± 7 GPa, indentation hardness *H*_IT_ = 4.9 ± 0.3 GPa, and density ρ = 2.63 g.cm^−3^. Nanoindentation used a Berkovich indenter at 50 mN, and density was determined by the Archimedes method. An apparent dissolution rate of 6.4 × 10^−4^ mm s^−1^ was estimated from photographic observations after 6 min of immersion in Hanks’ balanced salt solution [8]. This short-duration estimate documents rapid dissolution; it is not a standardized long-term corrosion rate or an in vivo resorption rate. The modulus and density motivate further implant-oriented research, whereas rapid dissolution and ribbon brittleness require substantial control of composition, microstructure, and processing.

Rapid solidification produces crystalline and glassy alloys within the Mg–Ca–Au system [8]. Work on Mg_82_Ca_8_Au_10_ has subsequently addressed atomic structure and devitrification in a glass-forming composition, identifying AuMg_3_ and an Mg-based solid solution containing Ca during crystallization [9]. The different Ca concentration and initial structural state make that phase sequence a comparison within the alloy family rather than a prediction for Mg_65_Ca_28_Au_7_. The present Ca-richer, initially crystalline ribbon offers a complementary route to identifying phases that can develop during processing. Established ternaries include CaAuMg_2_ with the MgCuAl_2_ type, CaAuMg with the TiNiSi type, and Ca_4_AuMg with the Gd_4_RhIn type [10,11,12]. Their distinct atomic arrangements make a phase-specific structural description necessary.

This study follows the sequence from melt-spun ribbon to a specimen thermally treated in a DSC instrument. Paired diffraction measurements reveal how the resolved crystalline assemblage changes, while the sharper pattern after treatment permits a positional refinement of CaAuMg_2_. Particular attention is given to the distinction between phase identification, conditional structural parameters, and approximate phase quantities. The objective is to provide a crystallographic foundation for later degradation and imaging studies of this alloy family. The materials question is how thermal processing changes the constituents available for phase-selective dissolution.

## 2. Materials and Methods

### 2.1. Ribbon Preparation and Experimental Sequence

**Figure 1:**
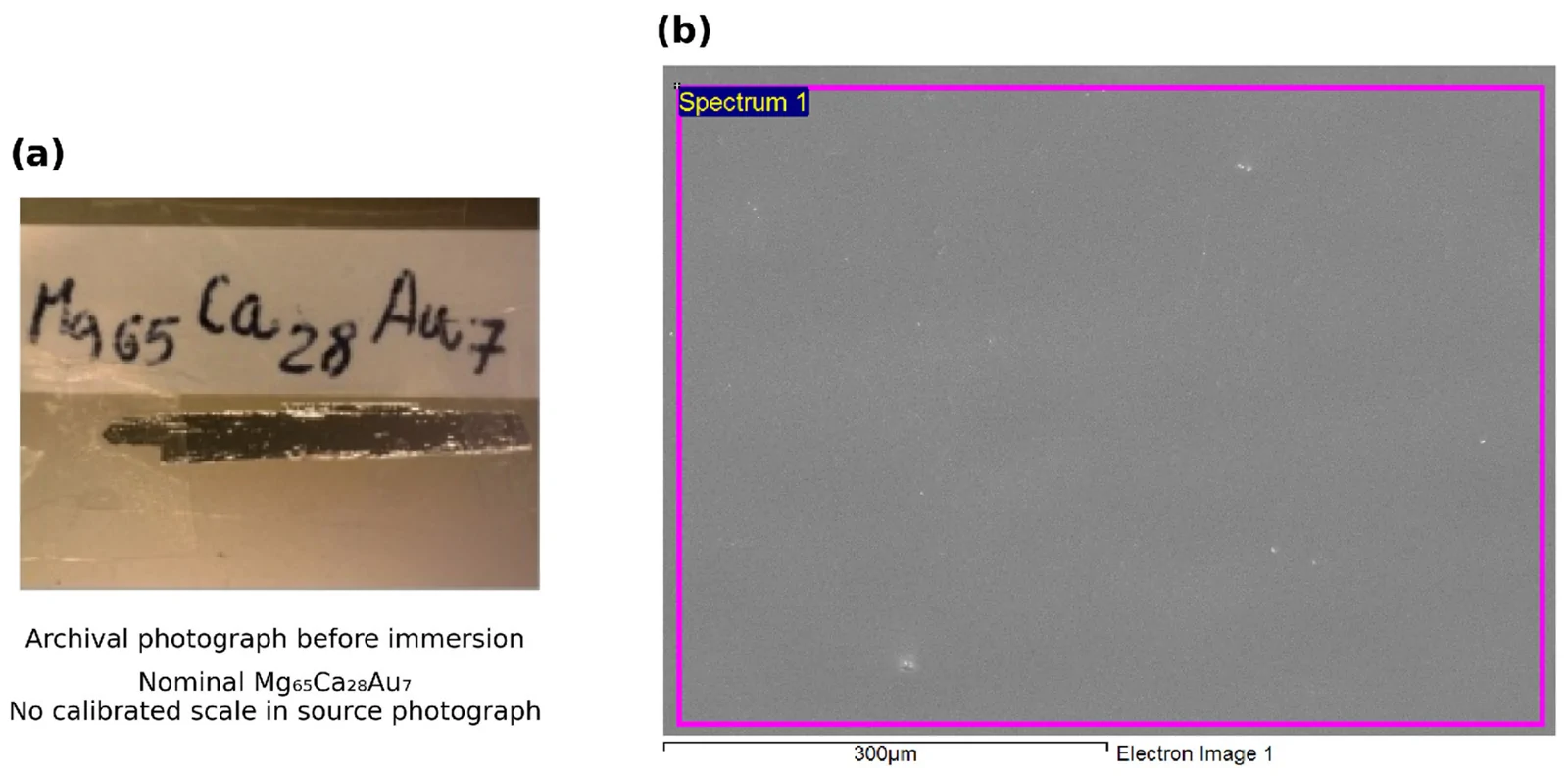
(**a**) Archival photograph of the nominal Mg_65_Ca_28_Au_7_ ribbon from Figure 3 in Ref. [8]. (**b**) SEM overview of the wheel-contact surface acquired with a JEOL JSM 7000F (JEOL Ltd., Tokyo, Japan). The outline marks the area of the EDX field, and the original 300 μm scale bar is retained.

The ribbon was prepared within the Mg–Ca–Au composition series reported in Ref. [8]. Elemental Ca, Mg, and Au, of purities 99.5, 99.98, and 99.99 wt.%, respectively, were combined to target Mg_65_Ca_28_Au_7_ in atomic percent. The constituents were induction-melted at 1000 °C in a quartz tube coated with boron nitride (BN), under a vacuum better than 3 × 10^−3^ Pa. The alloy was subsequently processed by the single-roller melt-spinning method to obtain rapidly solidified ribbons with a maximum thickness of 40 μm, a width of approximately 4 mm, and lengths below 70 mm. These dimensions describe the ribbon product and are not inferred from the uncalibrated archival photograph in Figure 1a. The nominal designation is retained for sample identification; the area-EDX estimate after preparation is reported separately in Section 3.1.

The experimental sequence was ribbon preparation, SEM/area EDX on the wheel-contact surface, XRD of the as-spun ribbon, thermal treatment during DSC, and XRD after the DSC cycle. Thus, the two diffraction patterns represent distinct processing states. The earlier Mg_64_Ca_27_Au_9_ designation refers to the rounded EDX composition associated with the nominal Mg_65_Ca_28_Au_7_ material. Comparisons with Ref. [8] concern this preparation series and composition. The laboratory patterns examined here are distinct from the high-energy diffraction measurements reported in Ref. [8].

### 2.2. SEM/EDX and the Scope of Electron Microscopy

The wheel-contact side of the ribbon was examined using a JEOL JSM 7000F scanning electron microscope (JEOL Ltd., Tokyo, Japan). No chemical etching or other etching treatment was applied prior to examination. The SEM observations were intended to characterize the surface morphology and identify the region selected for elemental analysis by EDX, rather than to determine grain morphology, grain size, or the spatial distribution of individual crystalline phases [13]. Two area analyses are reported individually together with their mean.

Transmission electron microscopy is especially demanding for this system because the ribbon is brittle, difficult to thin into an electron-transparent specimen, and contains Mg and Ca, which readily oxidize. These preparation constraints limit access to representative, minimally altered thin regions. No TEM results are therefore included in this study.

### 2.3. DSC Treatment

DSC was used as a controlled thermal treatment intended to promote further crystallization of the ribbon. A 4.9 mg specimen was measured in a PerkinElmer DSC 8500 (PerkinElmer, Inc., Waltham, MA, USA) using graphite pans, an argon atmosphere, and a programmed purge flow of 20 mL min^−1^. The program heated from 0 to 400 °C at 10 K min^−1^, followed by ballistic cooling to 0 °C. The heating signal is shown against recorded sample temperature without smoothing or baseline subtraction. The cooling export is largely constant and is not used to assign transformations. No transition enthalpies or onset temperatures were extracted.

### 2.4. X-Ray Diffraction and Refinement Strategy

Both patterns were collected on an X’Pert PRO diffractometer (PANalytical B.V., Almelo, The Netherlands) with a Cu anode at 40 kV and 50 mA, a fixed 0.5° divergence slit, and continuous gonio scanning. Acquisition parameters are summarized in Table 1. The Cu Kα doublet was represented by λ_1_ = 1.54056 Å and λ_2_ = 1.54439 Å with I_2_/I_1_ = 0.5. The entire recorded range of each pattern was retained, using Poisson weights w_i_ = 1/y_i_.

Rietveld calculations used GSAS-II release 5799 [14]. Reference Cu Kα powder patterns were calculated from the following crystallographic models: trigonal Mg_3_Au, P−3c1 (No. 165), COD 1510235, from Daams and van Vucht [15]; C14 Mg_2_Ca, P6_3_/mmc (No. 194), COD 4313238, from Amerioun et al. [16]; and CaAuMg_2_, Cmcm (No. 63), using the powder cell and structural assignment of Kersting et al. [10] with the MgCuAl_2_-type site arrangement [17]. The CaAuMg_2_ reference in Ref. [10] provides powder lattice parameters. Its single-crystal coordinates are reported for the isotypic SrPdMg_2_, not for CaAuMg_2_. The ternary positional-model provenance and the transferred-coordinate comparison are specified in Section 2.5. These calculated patterns, their peak positions and full-profile agreement underpin phase identification. The dominant as-spun reflections were modeled with Mg_2_Ca and CaAuMg_2_, while Mg_3_Au was included in separate trace-phase tests. The after-DSC refinement contained all three phases. Atomic coordinates of the binary phases were fixed. Four symmetry-independent coordinates of CaAuMg_2_ were refined against the after-DSC pattern and transferred as fixed values to the as-spun refinement. Occupancies were fixed at unity, and isotropic displacement parameters were held fixed. The ternary *U*_iso_ values were fixed at zero because a trial common displacement parameter was unresolved within uncertainty; this convention does not imply absent atomic motion.

The refinements included phase scales, lattice parameters, sample displacement, effective isotropic size terms, and preferred orientation. A March–Dollase correction along the crystallographic direction [001] was used for Mg_3_Au [18], and sixth-order spherical-harmonic corrections for Mg_2_Ca and CaAuMg_2_. All calculated reflection corrections remained positive. Instrument-profile and microstrain parameters were fixed. Smooth Chebyshev backgrounds contained 30 terms for the as-spun pattern and 20 for the after-DSC pattern, accommodating their different broad backgrounds. Effective size parameters describe peak profiles under these assumptions and are not independently calibrated crystallite sizes.

Mass fractions were calculated from phase scales and unit-cell masses and normalized over the modeled crystalline phases [19]. They exclude any unquantified non-Bragg-scattering component. Texture, absorption contrast between Au-bearing and Au-free phases, fixed stoichiometries, and different sampled volumes limit absolute quantification. Standard uncertainties are conditional least-squares estimates [20]. Profile plots, background-subtracted residuals, and sensitivity tests were considered alongside conventional agreement factors [21]. The EDX composition was also compared with the elemental proportions reconstructed from the normalized phase fractions and ideal phase formulae.

### 2.5. Geometry, Visualization and Crystallographic Files

Periodic neighbors were generated from the space-group operations and refined lattice metrics. Conditional uncertainties of selected CaAuMg_2_ distances were propagated from the covariance matrix, including positional and lattice correlations. The comparison geometry of Kersting et al. was reconstructed from their CaAuMg_2_ powder cell and SrPdMg_2_ fractional coordinates, following the construction underlying their tabulated contacts [10]. Shaded sphere-and-cylinder renderings were generated directly from crystallographic coordinates. Atom colors and radii are illustrative; connectors indicate geometric contacts rather than bond orders. CIF exports include symmetry operations, site multiplicities, refinement metadata, and the status of fixed or refined parameters.

## 3. Results and Discussion

### 3.1. Composition of the Ribbon

The two area EDX analyses gave closely similar local estimates (Table 2), with an arithmetic mean of 64.43 at.% Mg, 26.86 at.% Ca, and 8.73 at.% Au. The corresponding EDX estimate of the mean Au content is about 39.4 wt.%; thus, its minority atomic fraction should not be mistaken for a small mass fraction. The rounded Mg_64_Ca_27_Au_9_ designation in Ref. [8] is consistent with these measurements. Figure 1a illustrates the ribbon form using the archival pre-immersion photograph of the nominal Mg_65_Ca_28_Au_7_ specimen, and Figure 1b shows the representative EDX field.

### 3.2. Evolution of the Crystalline Phase Assemblage

**Figure 2:**
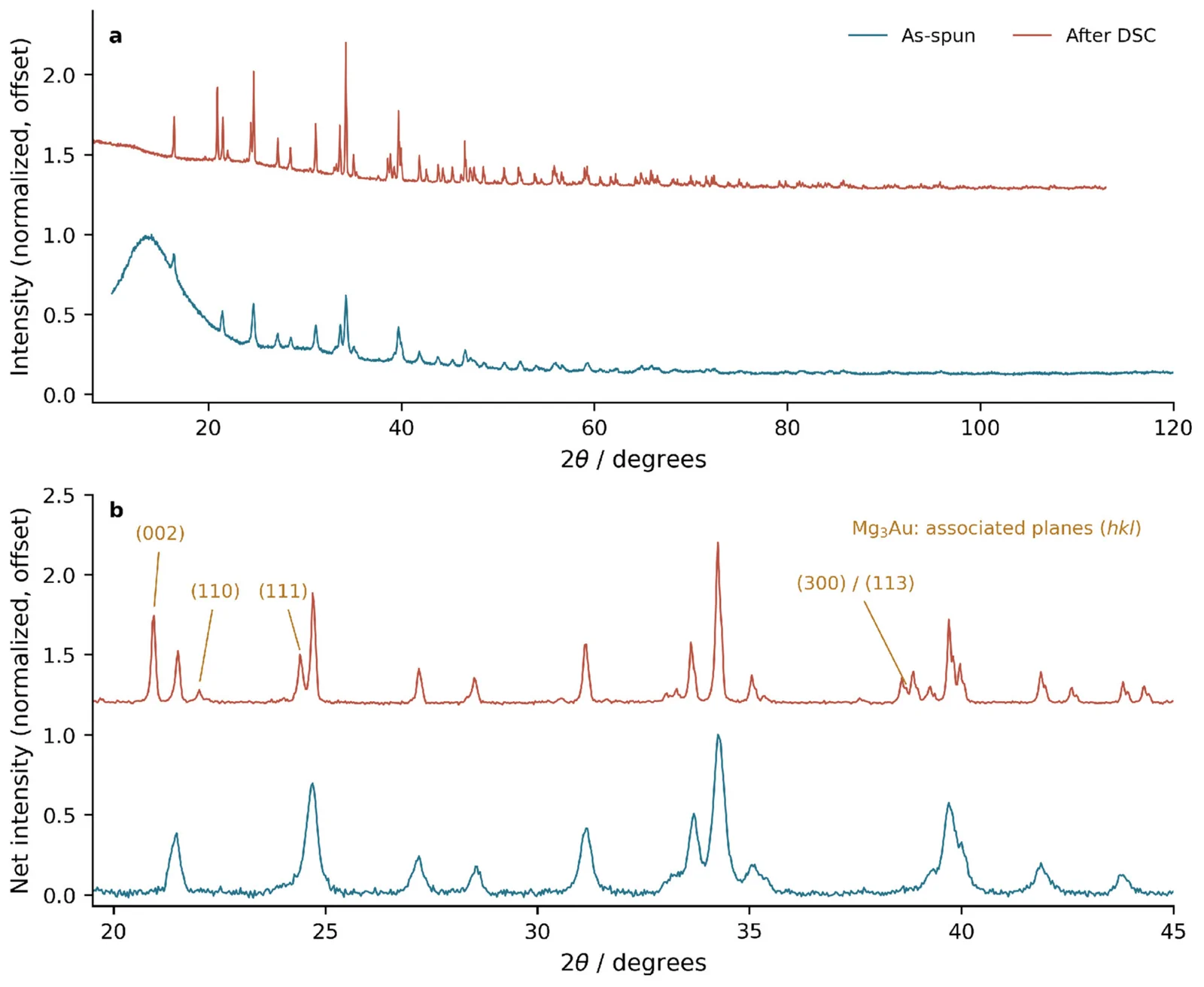
Direct comparison of XRD before and after annealing in the DSC. (**a**) Complete measured patterns, each divided by its own maximum measured intensity. (**b**) Expanded interval after subtracting the fitted background and dividing by each pattern’s maximum net Bragg intensity. The after-DSC curves are offset by 1.2. Selected Mg_3_Au peaks are labeled by their associated lattice planes.

The paired patterns show corresponding reflections from Mg_2_Ca and CaAuMg_2_ before and after treatment (Figure 2). The as-spun state additionally has a pronounced broad low-angle contribution and wider Bragg peaks. After annealing in the DSC, the crystalline reflections are sharper, and Mg_3_Au peaks are clearly resolved, notably the peaks associated with the (002) planes near 2θ = 20.95°, (110) near 22.02°, (111) near 24.42°, and the overlapping (300)/(113) contributions near 38.61–38.87°. These changes demonstrate evolution of the resolved crystalline assemblage and increased diffraction coherence during the thermal cycle.

The two-phase description of the as-spun pattern gives approximately 56.9 wt.% Mg_2_Ca and 43.1 wt.% CaAuMg_2_ within the modeled crystalline fraction. Adding Mg_3_Au with fixed effective size parameters of 0.05 or 0.5 μm gives only 0.38 or 0.19 wt.%, respectively, while *R*_wp_ changes from 2.0555% to 2.0517% or 2.0529%. These small, profile-dependent improvements show that Mg_3_Au is not securely distinguishable under the selected background and peak-profile treatment; they do not establish its absence. Broad Mg_3_Au reflections could overlap the tails of stronger peaks or be absorbed into the fitted broad background. The fitted trace fractions are therefore neither detection limits nor upper bounds on a poorly crystalline Mg_3_Au component. The two-phase fit describes the constituents securely resolved by the present data, rather than an exhaustive inventory of the as-spun material.

After DSC, the refined crystalline proportions are 51.8 wt.% Mg_2_Ca, 37.8 wt.% CaAuMg_2_, and 10.5 wt.% Mg_3_Au (Table 3). The principal observation is the marked increase in the resolved Mg_3_Au contribution, rather than proof of its first nucleation during heating. Its appearance could involve crystallization of previously poorly ordered material, growth of a small pre-existing fraction, or redistribution between constituents. Ex situ diffraction does not distinguish these pathways or determine a transformation temperature. Likewise, the lower normalized CaAuMg_2_ fraction after DSC cannot by itself establish consumption of the ternary phase, because the denominator changes and the absolute crystalline fraction is unknown.

The resolved Mg_3_Au contribution has a useful comparison in the crystallization of Mg_82_Ca_8_Au_10_ glass, where AuMg_3_ was reported together with an Mg-based solid solution containing Ca [9]. Here, the substantially higher nominal Ca content accompanies an assemblage containing Mg_2_Ca and CaAuMg_2_ in both measured states. The common Au–Mg binary constituent supports comparison within the alloy family, while the different starting structures and Ca contents prevent transferring a crystallization sequence or transformation temperature from that glass to this ribbon.

**Figure 3:**
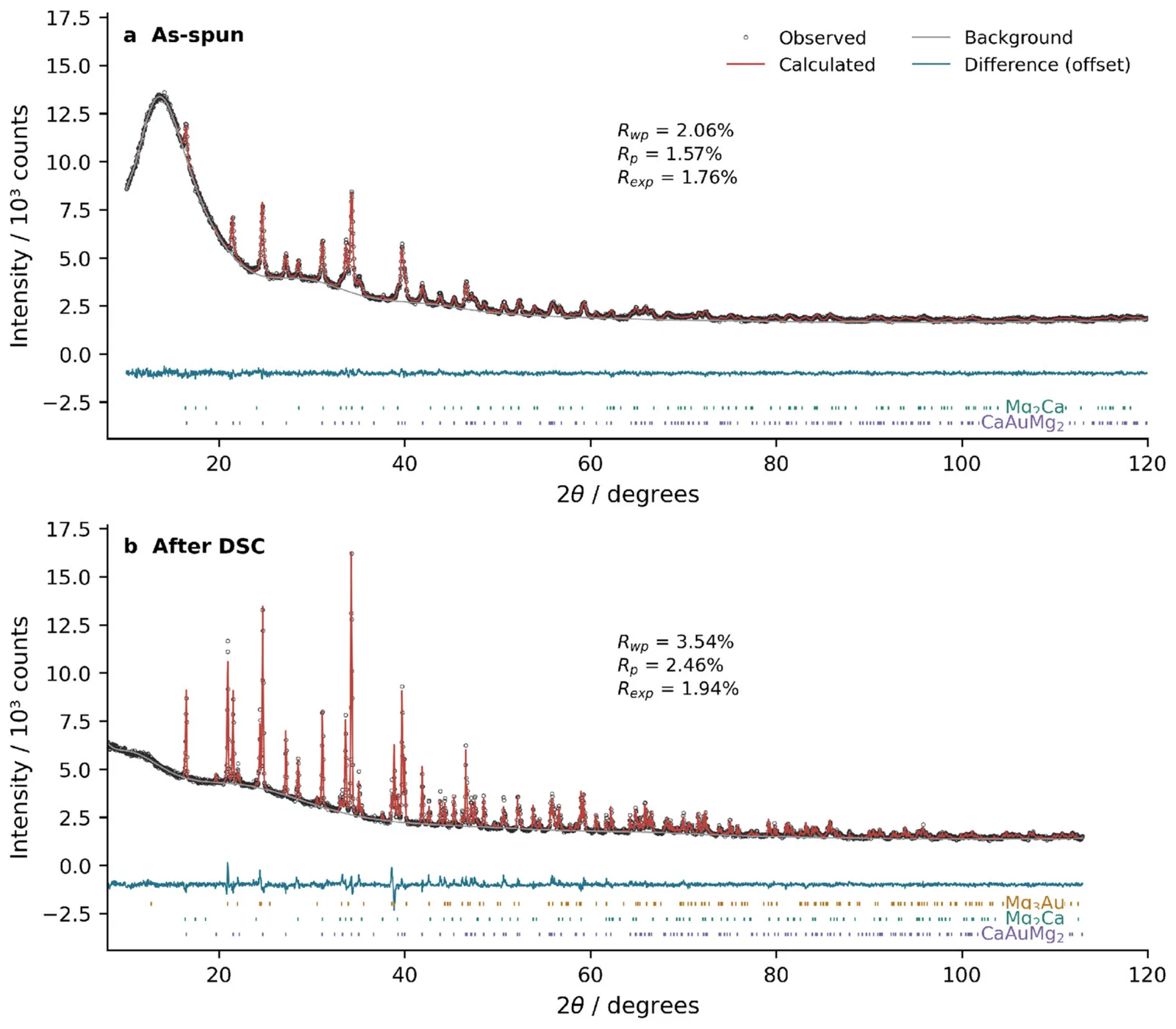
Rietveld refinements of (**a**) the as-spun ribbon and (**b**) the ribbon after DSC annealing. Observed points, calculated profiles, backgrounds, and offset difference curves are shown over the complete measured ranges. Reflection rows are labeled by phase. The unoccupied Mg_3_Au row in panel a is omitted. Enlarged net-profile residuals are included in the Supplementary Materials.

The Rietveld fits give *R*_wp_ = 2.06% and 3.54% for the as-spun and after DSC annealing states, respectively (Figure 3). The differing count statistics and backgrounds prevent ranking the two datasets by *R*_wp_ alone, consistent with the cautions discussed by Toby [21]. When normalized to the background-subtracted signal, the corresponding weighted residuals are 17.2% and 18.7%, revealing the remaining Bragg-profile mismatch. These auxiliary values are background-dependent; their purpose is to expose the Bragg-profile mismatch that a small whole-pattern *R_wp_* can conceal.

The changes in fitted cells are small but resolved within the formal model: CaAuMg_2_ changes from a = 4.3090(3), b = 10.7455(9), c = 8.2606(6) Å to a = 4.3121(1), b = 10.7642(2), c = 8.2596(2) Å. The approximately 0.23% increase in its cell volume accompanies peak sharpening. Such changes can be consistent with relaxation of a rapidly solidified microstructure, but specimen effects and possible compositional redistribution remain alternative contributions. Peak narrowing can reflect larger coherently diffracting domains and/or reduced microstrain [22]. Because instrumental-profile and microstrain terms were fixed here, these effects were not independently separated. Consequently, neither the effective size parameters nor the small cell shifts establish a unique relaxation mechanism or a change in phase stoichiometry [20].

### 3.3. Role of the DSC Annealing

**Figure 4:**
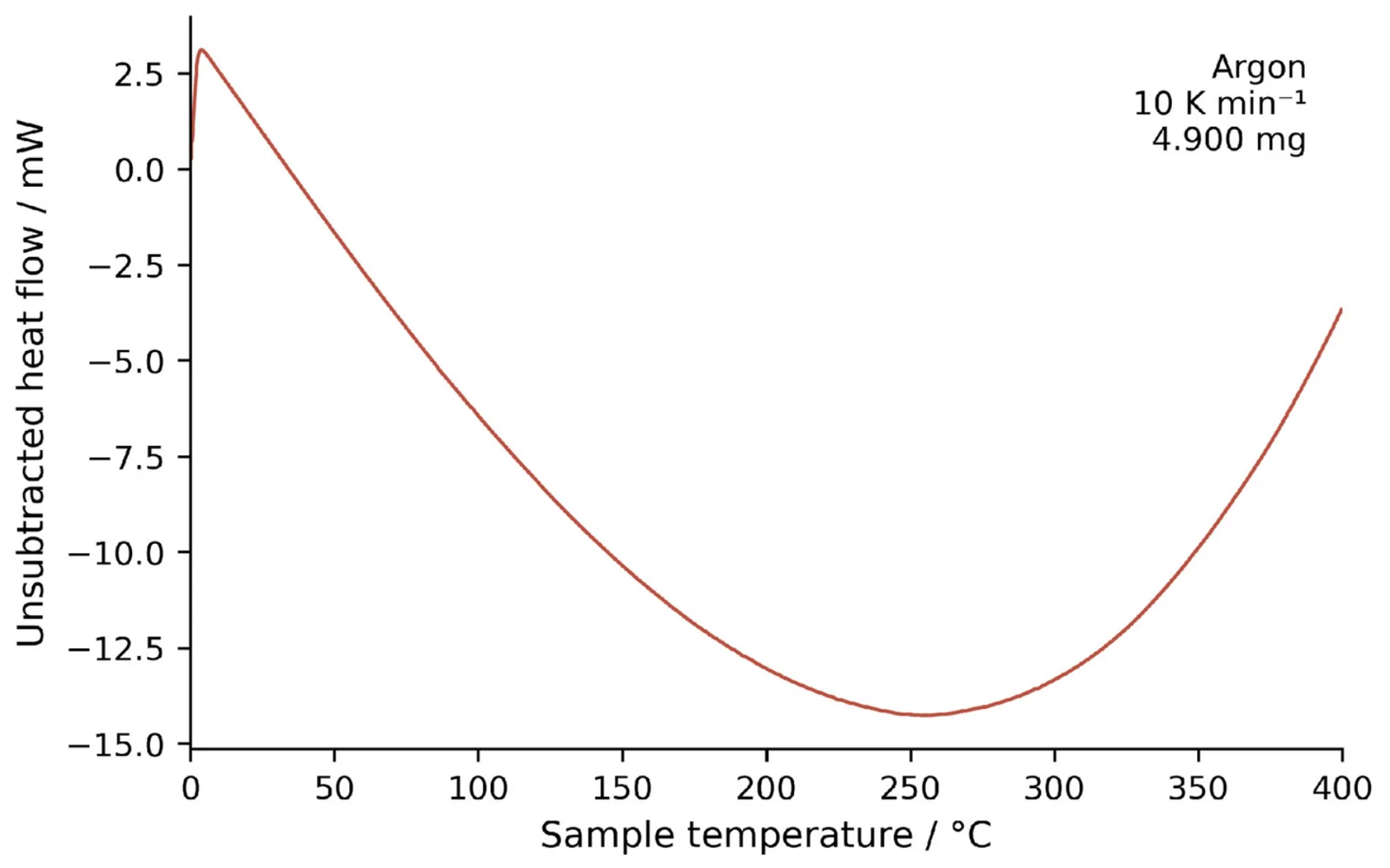
DSC heating trace in argon for the 4.900 mg specimen, heated at 10 K min^−1^ to approximately 400 °C. The recorded, unsubtracted heat-flow signal is shown without smoothing. No significant localized transformation peak is resolved; no transition temperature or enthalpy is assigned.

The DSC heating trace contains no clearly resolved, significant localized signature of a phase transition (Figure 4). Its smooth curvature is not assigned to a crystallization peak. Nevertheless, the paired XRD patterns show that the thermal cycle changes the resolved phase assemblage and peak widths. Thus, treatment intended to increase crystallization need not produce a separately resolved calorimetric event in this measurement. The absence of such a signal does not establish phase invariance or an equilibrium stability limit.

### 3.4. Refinement and Structural Context of CaAuMg_2_

The ternary phase remains a substantial crystalline constituent in both states. Its Cmcm symmetry, Z = 4, and MgCuAl_2_-type site topology distinguish it from the other known Ca–Au–Mg ternaries. After DSC, the refined cell closely matches the CaAuMg_2_ powder cell reported by Kersting et al., a = 4.3127(6), b = 10.7530(10), c = 8.2620(10) Å [10]. The relative differences are −0.015%, +0.104% and −0.030%, respectively. This agreement supports identification with the known compound.

The additional contribution of the present analysis is a powder-based refinement of the four independent positional parameters: y(Mg), z(Mg), y(Au) and y(Ca), with Mg on 8f and Au and Ca on 4c (Table 4). Releasing these parameters lowers *R*_wp_ from 3.650% to 3.542% under the same wavelength and profile assumptions. Starting from the SrPdMg_2_ coordinates used as a literature reference converges to positions within 0.00011 in fractional coordinates of the reported solution. The binary coordinates and all occupancies remain fixed. This is a constrained multiphase powder determination, rather than a complete refinement of composition and atomic displacement parameters.

Preferred orientation changes relative intensities, so its treatment can couple to atomic coordinates through the calculated structure factors [18,20]. Texture is therefore a material limitation on the positional interpretation. Lowering the ternary spherical-harmonic order from six to four increases *R*_wp_ to 3.879% and shifts z(Mg) by approximately 0.0105, exceeding its conditional uncertainty in the selected model. A common ternary *U*_iso_ refines to 0.00017(73) Å^2^ and provides no resolved displacement information. The robust result is the Cmcm phase assignment and its lattice metric. The positions provide a reproducible working structural description, while fine differences in Mg coordination require confirmation with more nearly randomized or higher-resolution diffraction data.

**Figure 5:**
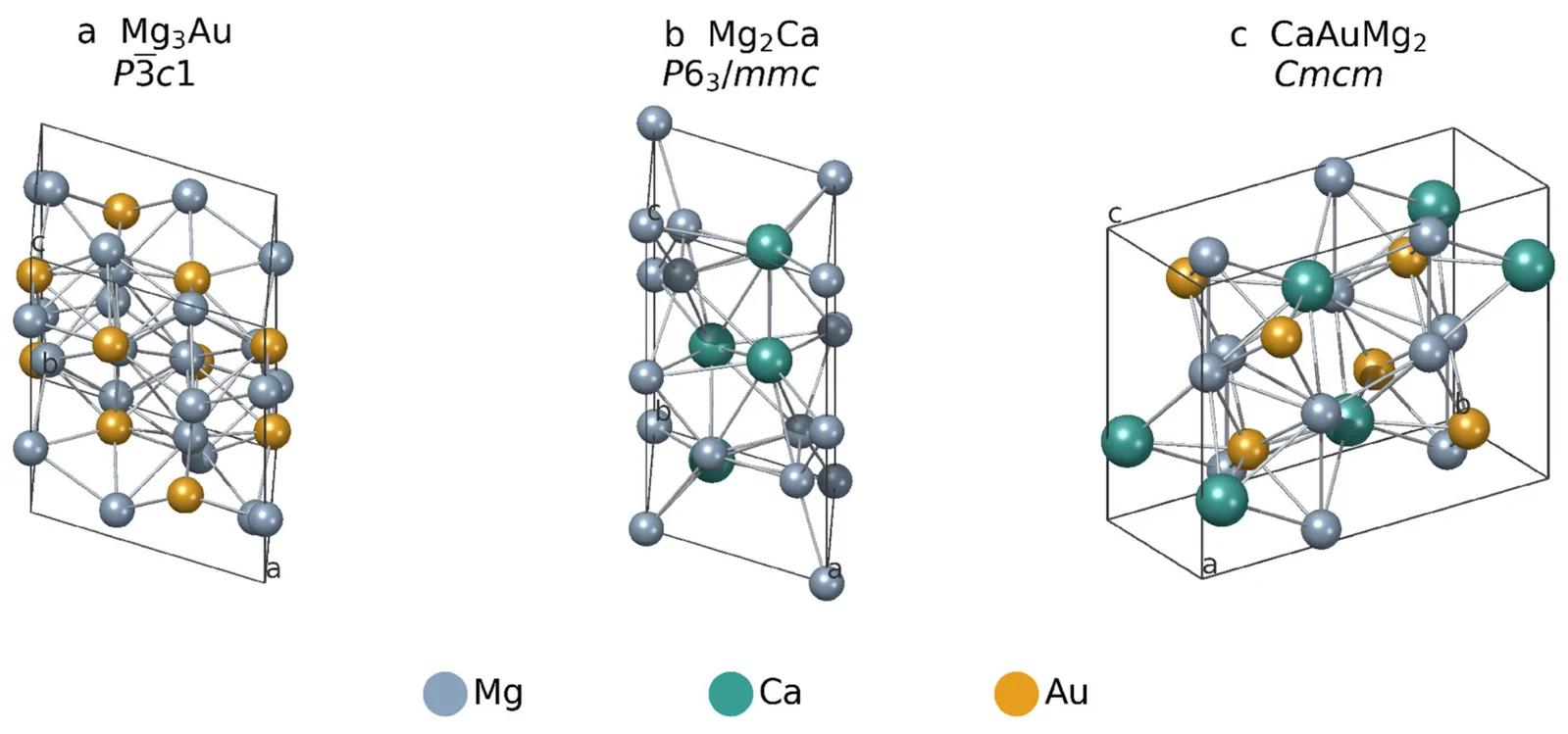
Shaded crystallographic renderings of (**a**) Mg_3_Au, P−3c1, (**b**) C14 Mg_2_Ca, P6_3_/mmc, and (**c**) CaAuMg_2_, Cmcm. Mg, Ca, and Au are blue-gray, green, and gold, respectively. Unit-cell edges and lattice-vector endpoints are shown.

Figure 5 visualizes all identified phases after the DSC annealing. C14 Mg_2_Ca forms a Laves framework, Mg_3_Au has a trigonal structure with several inequivalent Mg sites, and CaAuMg_2_ separates large Ca coordination cavities from the Au–Mg framework [10,15,16]. The structures are chemically and geometrically distinct, so the response of the alloy during degradation cannot be inferred from the nominal composition alone.

**Figure 6:**
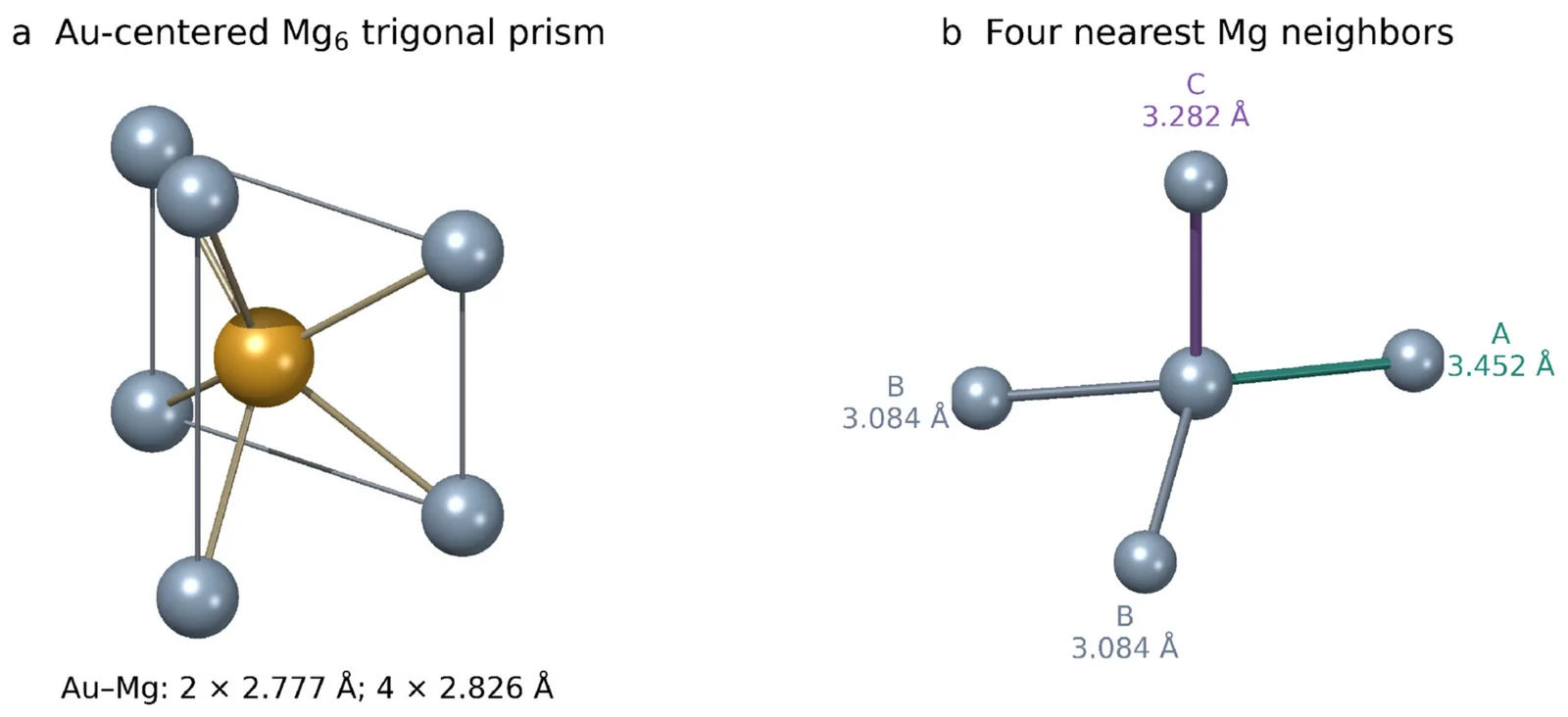
Principal local motifs of the after-DSC CaAuMg_2_ model: (**a**) Au-centered Mg_6_ prism and (**b**) the four nearest Mg neighbors around a central Mg. A, B, and C label the contact families compared with Ref. [10]; B occurs twice. Distances in the image are calculated values; their conditional uncertainties are given in the text. The connectors and photorealistic shading visualize geometry and do not measure bond order or electron density.

The essential local motif of CaAuMg_2_ is an Au-centered Mg_6_ trigonal prism, with two Au–Mg distances of 2.777(12) Å and four of 2.826(7) Å (Figure 6). Four nearest Mg neighbors define contacts A = 3.452(23) Å, B = 3.084(17) Å (twice) and C = 3.282(23) Å. The geometry reconstructed from the CaAuMg_2_ cell and SrPdMg_2_ coordinates in Ref. [10] instead gives approximately 3.674, 2.999, and 3.147 Å. That publication determined single-crystal coordinates for SrPdMg_2_ and SrPdCd_2_, not CaAuMg_2_ itself. The present comparison therefore tests a transferred-coordinate construction. It supports the lonsdaleite-related, distorted Mg connectivity, but the texture sensitivity precludes assigning a new bonding regime from these distance differences.

Kersting et al. supported their bonding discussion with electronic-structure and overlap-population calculations for SrPdMg_2_ and SrPdCd_2_ [10]. Those calculations concern different chemistries and cannot establish bond strengths in CaAuMg_2_. The present contribution is the experimentally constrained geometry of the Au-containing member, with explicit sensitivity to texture.

CaAuMg and Ca_4_AuMg differ from CaAuMg_2_ in stoichiometry, symmetry, and connectivity (Table 5). In particular, the isolated Mg_4_ tetrahedra reported for the Ca-rich Gd_4_RhIn family [12] contrast with the extended Mg substructure of CaAuMg_2_ [10]. CaAuMg instead has the ordered TiNiSi-type framework and a different Au:Mg ratio [11]. The present model does not require either alternative ternary as a resolved constituent. This conclusion concerns the measured ribbon states and is not an exhaustive determination of the equilibrium Mg–Ca–Au phase diagram.

### 3.5. Compositional Constraints and Relation to Earlier Work

Ref. [8] was a composition-series screening study of glass formation, indentation properties, density, and short-duration dissolution. Its diffraction results were presented as total structure factors and pair-distribution functions, and Table 1 classified the Mg_64_Ca_27_Au_9_ specimen as crystalline (C). Individual CaAuMg_2_ or Mg_3_Au phase assignments and Rietveld phase fractions were not reported for that specimen. The present work therefore extends the earlier crystalline-state classification by identifying resolved constituents and comparing two processing states; it does not replace a contradictory named-phase assignment in that publication. The earlier synchrotron experiment at Diamond I15-1 used 76.69 keV radiation (λ = 0.16166 Å) in transmission, whereas the present laboratory measurements use Cu Kα radiation. Intensities and backgrounds from these different geometries and sampled volumes are not directly interchangeable.

The modulus, hardness, density, and apparent dissolution estimate quoted from Ref. [8] characterize the rapidly solidified material of that composition series. They provide context for the present as-spun state, but they are not newly measured properties and cannot be assigned to the DSC-treated state. The photographic dissolution estimate was not phase-resolved. It therefore cannot establish whether CaAuMg_2_ or Mg_3_Au controls the initial attack, nor can it show that the thermal cycle improved corrosion resistance. Such a comparison requires matched specimens in both states, identical exposure conditions, and measurements of degradation and retained mechanical integrity.

**Figure 7:**
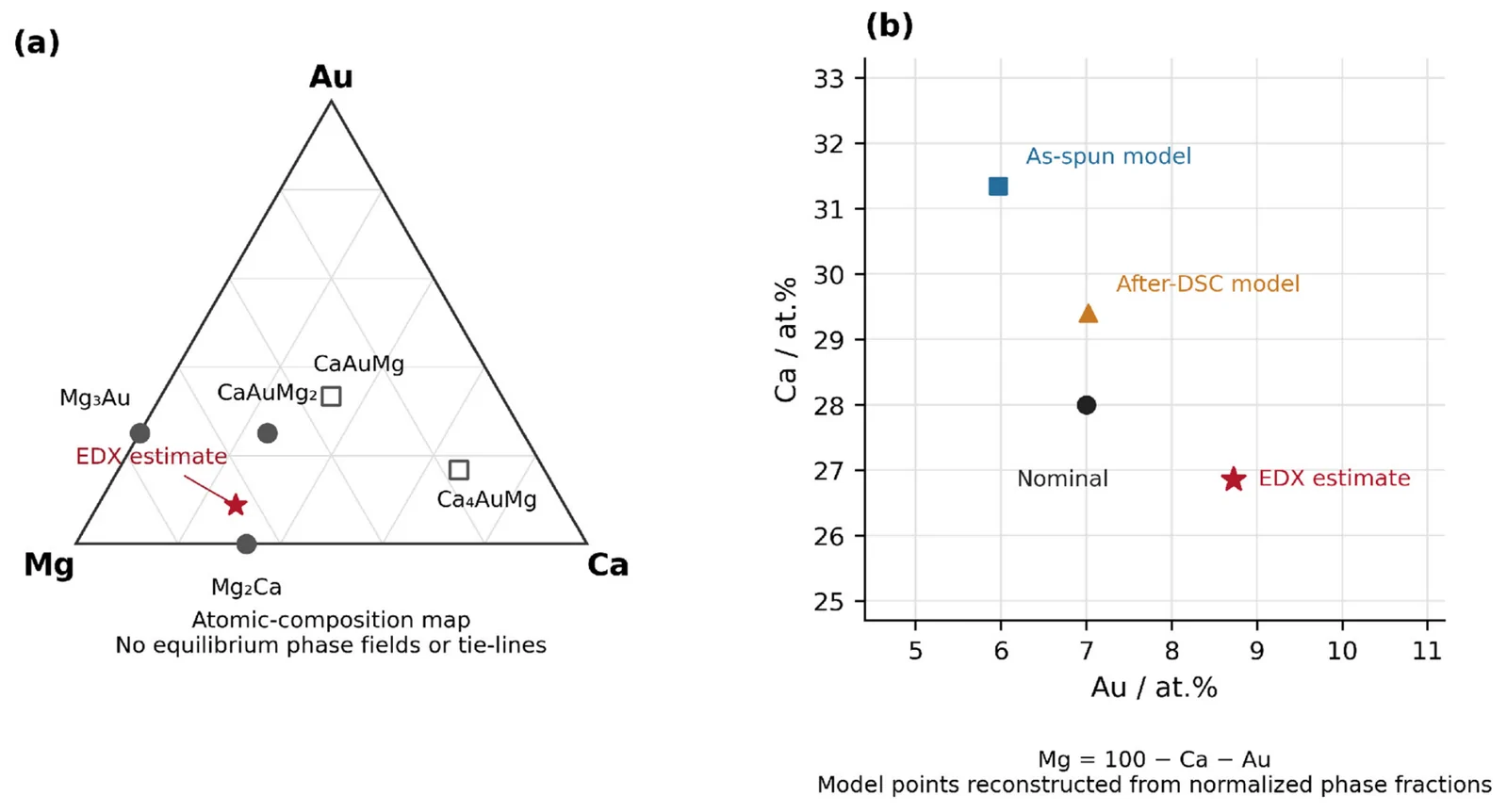
(**a**) Atomic-composition map of Mg–Ca–Au showing the semiquantitative area-EDX estimate, the identified phase stoichiometries (filled circles) and the comparison ternaries CaAuMg and Ca_4_AuMg (open squares). (**b**) Comparison of the nominal composition, the normalized mean area-EDX estimate, and elemental proportions reconstructed from the normalized Rietveld fractions. The model points are calculated consistency checks, not independent composition measurements; Mg = 100 − Ca − Au.

Figure 7a places the area-EDX composition estimate and the relevant known compounds on an atomic-composition triangle. It is a composition map, not an equilibrium phase diagram. A recent thermodynamic optimization by Gierlotka et al. describes the binary Au–Mg system [23]; it does not supply the ternary interactions or equilibria needed to predict the present assemblage. No validated Mg–Ca–Au isothermal section applicable to this composition and treatment was identified in the literature examined here. Moreover, melt spinning and a continuous DSC cycle do not establish equilibrium. Phase predictions based solely on the binary edges or on the glass-forming map would therefore exceed the available evidence.

The composition comparison also constrains quantitative interpretation (Figure 7b). Converting the normalized phase mass fractions to moles of each element using the ideal phase formulae gives approximately 62.7 at.% Mg, 31.3 at.% Ca and 6.0 at.% Au for the as-spun model, and 63.6 at.% Mg, 29.4 at.% Ca and 7.0 at.% Au for the after-DSC model. These point estimates differ from the area-EDX mean of approximately 64.4 at.% Mg, 26.9 at.% Ca and 8.7 at.% Au. The EDX result is itself semiquantitative [13], so the discrepancy does not define the error of either method or establish a missing phase. Texture and absorption effects, unmodeled poorly ordered material, fixed phase stoichiometries, and different sampled volumes can contribute to this comparison [19,20], but their individual contributions have not been separated.

### 3.6. Implications for Biodegradable Alloy Design

The phase evolution establishes which crystalline constituents must be considered when connecting processing to degradation. Au is partitioned into at least CaAuMg_2_ and, after the DSC annealing, Mg_3_Au, while Mg_2_Ca remains the largest modeled mass fraction. The Mg–Ca studies of Li et al. [2] and Jia et al. [5] show that the presence and distribution of Mg_2_Ca can affect degradation. This supports testing phase-selective dissolution in the present alloy, but it does not establish the electrochemical role of its Au-bearing phases. Their individual degradation kinetics have not been measured here. Future experiments should relate the identified phases to mechanical retention, Au-containing residues, and CT visibility during exposure to physiological media. The earlier hardness, modulus, and density values characterize the rapidly solidified alloy and should not be assigned unchanged to the DSC-treated state. Phase distribution and local electrochemical interactions, discussed for Mg-based biomaterials in Ref. [4], motivate examining whether selective removal of a connected constituent interrupts the remaining load-bearing framework. The Au-containing compounds should not automatically be assigned the electrochemical behavior of elemental Au. Their potentials and dissolution products require direct measurements. Consequently, increased diffraction coherence after DSC is a structural observation, not evidence of slower degradation.

The next discriminating experiments are matched immersion or electrochemical tests on as-spun and DSC-treated ribbons, combined with spatially resolved chemical analysis of attack sites and residues. EBSD or higher-resolution electron microscopy could test phase distribution and orientation if a representative surface can be prepared without appreciable oxidation or preparation damage. These measurements would connect the present phase identifications to corrosion localization and mechanical retention. Until then, the low modulus and potential radiopacity remain design motivations rather than validated implant performance.

## 4. Conclusions

Paired diffraction measurements establish an evolution from an as-spun ribbon dominated by crystalline Mg_2_Ca and CaAuMg_2_ to a sharper three-phase assemblage containing clearly resolved Mg_3_Au after DSC treatment in argon. The approximate normalized crystalline fractions change from 56.9 wt.% Mg_2_Ca and 43.1 wt.% CaAuMg_2_ to 51.8 wt.% Mg_2_Ca, 37.8 wt.% CaAuMg_2_ and 10.5 wt.% Mg_3_Au. Mg_3_Au is not securely resolved in the as-spun pattern. No significant localized phase-transition signature is visible in the DSC heating trace, so the structural evolution is established by the paired XRD results.

The after-DSC data support refinement of the CaAuMg_2_ cell and four independent atomic coordinates within the known Cmcm, MgCuAl_2_-type structure. AuMg_6_ prisms and distorted Mg connectivity provide the essential local description. Texture sensitivity limits interpretation of fine positional differences. The accompanying CIFs make these phase models reusable and suitable for prospective submission to public crystallographic databases such as COD [24], with their refinement constraints and provenance retained.

The relatively low modulus and density, together with the potential radiopacity introduced by Au, justify further implant-oriented development. If dissolution can be slowed and fracture resistance improved, this alloy family could be explored for temporary low-load bearing fixation elements, such as small pins for small-bone or craniofacial repair, or for resorbable interfacial layers. These are development targets rather than demonstrated applications of the present brittle, rapidly dissolving ribbon. By identifying the resolved constituents and their response to thermal treatment, this study defines phase-specific questions for subsequent degradation, mechanical-retention, and imaging experiments.

## Figures and Tables

**Table 1:** Laboratory diffraction datasets and final refinement statistics. *R*_exp_ is calculated from *R*_wp_/GOF; GOF = √χ^2^_r_.

Parameter	As-Spun	After DSC
2θ range/°	10–120	8–113
Nominal step/°	0.03342	0.03342
Recorded time per step/s	45	45
Number of observations	3291	3142
Independent refined parameters	53	52
*R*_wp_/%	2.056	3.542
*R*_p_/%	1.568	2.465
*R*_exp_/%	1.757	1.942
GOF	1.170	1.823

**Table 2:** Nominal composition and semiquantitative area-EDX estimates of the ribbon in atomic percent, normalized over Mg, Ca and Au. The mean is calculated from the two listed areas; rounding can cause totals to differ slightly from 100%. Digits are retained from the exported values and their arithmetic mean; they do not represent validated bulk-composition accuracy. Agreement between two areas is not a full estimate of EDX accuracy or bulk homogeneity.

Composition	Mg/at.%	Ca/at.%	Au/at.%
Nominal	65	28	7
Area 1	64.46	26.78	8.77
Area 2	64.39	26.93	8.68
Area mean	64.43	26.86	8.73

**Table 3:** Refined lattice parameters and normalized crystalline mass fractions. Parentheses give conditional standard uncertainties. Hexagonal settings have b = a and γ = 120°; the Cmcm cell is orthorhombic. Fractions are approximate and do not include systematic errors.

Phase	State	a/Å	b/Å	c/Å	V/Å^3^	wt.%
Mg_2_Ca	As-spun	6.2455(12)	a	10.1389(15)	342.50(9)	56.9
CaAuMg_2_	As-spun	4.3090(3)	10.7455(9)	8.2606(6)	382.49(6)	43.1
Mg_2_Ca	After DSC	6.2579(4)	a	10.1527(5)	344.326(25)	51.8
CaAuMg_2_	After DSC	4.3121(1)	10.7642(2)	8.2596(2)	383.374(13)	37.8
Mg_3_Au	After DSC	8.0765(8)	a	8.4831(3)	479.21(5)	10.5

**Table 4:** Atomic positions of CaAuMg_2_ refined from the after-DSC pattern in Cmcm. Occupancies are unity and *U*_iso_ = 0 Å^2^ is fixed. Only y(Mg), z(Mg), y(Au), and y(Ca) are independent; uncertainties are conditional. These coordinates were transferred and fixed in the as-spun fit.

Atom	Wyckoff Site	x	y	z
Mg1	8f	0	0.3446(11)	0.0513(14)
Au1	4c	0	0.76990(21)	¼
Ca1	4c	0	0.0579(9)	¼

**Table 5:** Structural comparison with established Ca–Au–Mg ternaries. The other compounds provide context; they are not identified as resolved constituents of the present patterns.

Compound	Structure/Space Group	Principal Distinction	Reference
CaAuMg_2_	MgCuAl_2_/Cmcm	AuMg_6_ prisms; distorted, extended Mg substructure; Au:Mg = 1:2	[10]
CaAuMg	TiNiSi/Pnma	Different ordered ternary framework; Au:Mg = 1:1	[11]
Ca_4_AuMg	Gd_4_RhIn/F − 43m	Isolated Mg_4_ tetrahedra and Au-centered Ca_6_ prisms	[12]

## Data Availability

The original contributions presented in this study are included in the article/Supplementary Materials. Further inquiries can be directed to the corresponding author.

## Supplementary Materials

The following supporting information can be downloaded at: https://www.mdpi.com/article/10.3390/ma19214654/s1, Supporting information comprises the refinement sensitivity analysis (Tables S1–S3), the net-profile residual plot (Figure S1), two GSAS-II 5799 projects and listings, five CIFs, measured XRD and DSC data, refined profiles, and reproducible plotting code.
