# Supplementary material for: Crystal Chemistry of a Biodegradable Mg65Ca28Au7 Alloy: Thermal Evolution and Refinement of CaAuMg2
Source: Materials (Basel). 2026 Oct 31;19(21):4654. doi: 10.3390/ma19214654 (PMC13764697; doi:10.3390/ma19214654)
Supplement: Supplementary file 1 [file materials-19-04654-s001.zip › materials_4612901_Supplementary_revised_red.pdf]

Supporting information

Crystal Chemistry of a Biodegradable Alloy with Nominal Composition $Mg_{65}Ca_{28}Au_7$: Thermal Evolution and Refinement of $CaAuMg_2$

Karel Saksl, Zuzana Molčanová, Beáta Ballóková and Renáta Chromá

Tables S1–S3 and Figure S1 document the existing refinement sensitivity analysis. Sections S7 and S8 add statistical and reflection-overlap diagnostics calculated from the same archived profiles and models. New and revised passages are shown in red. Numerical references in ordinary brackets refer to the main manuscript; supplementary references [S1] and [S2] are listed at the end.

S1 Data and refinement conventions

The as-spun XRD file is data/XRD_as_spun_original.csv; the after-DSC file is data/XRD_after_DSC_original.csv. The original CSV headers are retained. Profile exports contain 2θ, observed intensity, weight, calculated intensity, background, and observed-minus-calculated intensity. Poisson weights are 1/y. Both full measured angular ranges are included, with no excluded intervals. The Cu Kα wavelengths are 1.54056 and 1.54439 Å, and I_2/I_1 = 0.5.

Final projects in models/ embed the measured profiles and refinement controls. Each project has a matching native listing. The selected after-DSC refinement releases four independent $CaAuMg_2$ coordinates; these positions are transferred and fixed in the as-spun refinement. The binary coordinates and all occupancies are fixed. The ternary isotropic displacement parameters are fixed at zero because their trial common value is unresolved.

Crystalline phase fractions are normalized over the modeled phases. No internal standard, instrumental broadening standard, or microabsorption correction is available. Texture and absorption contrast therefore limit absolute phase quantities. The large background contribution is not an amorphous mass fraction.

S2 Sensitivity analysis

In Tables S1 and S2, p denotes the number of independent fit parameters.

Table S1. Tests of a possible Mg_3Au contribution in the as-spun pattern.

Model	p	Rwp (%)	GOF	Mg_3Au (normalized wt.%)
Selected two-phase model	53	2.055517	1.170167	Not included
Mg_3Au with fixed 0.05 µm effective size	54	2.051740	1.168197	0.375823
Mg_3Au with fixed 0.5 µm effective size	54	2.052881	1.168847	0.194471

The trace contribution changes with the imposed peak width and changes Rwp by less than 0.004 percentage points. These tests do not establish a secure initial Mg_3Au assignment or a formal detection limit. Its clear resolution after treatment need not mark its first nucleation.

Table S2. After-DSC ternary-model sensitivity.

Test	p	Rwp (%)	GOF	y(Mg)	z(Mg)	y(Au)	y(Ca)

Test	p	Rwp (%)	GOF	y(Mg)	z(Mg)	y(Au)	y(Ca)
Fixed coordinates	48	3.649971	1.877895	0.35930	0.05791	0.77041	0.05320
Refined positions SH6	52	3.541954	1.823500	0.34456	0.05135	0.76990	0.05793
Ternary texture SH4	48	3.879184	1.995824	0.34357	0.06185	0.77011	0.05598
Common Uiso trial	53	3.541877	1.823755	0.34441	0.05125	0.76990	0.05794
$SrPdMg_2$ restart	52	3.542007	1.823527	0.34445	0.05130	0.76990	0.05794

The selected four-coordinate refinement lowers Rwp from 3.64997% to 3.54195%. The alternative starting coordinates converge within 0.00011 in fractional coordinates. Reducing the ternary texture order shifts z(Mg) by about 0.0105; this exceeds its conditional standard uncertainty. A common Uiso of 0.00017(73) Å² is unresolved. The formal uncertainties do not encompass these systematic model dependencies.

S3 Agreement factors and background

The weighted residual sum is $S = \Sigma w_i(y_i - y_{calc,i})^2$. Conventional Rwp is calculated from the full signal. The auxiliary net-profile value uses $\Sigma w_i(y_i - b_i)^2$ in the denominator instead of $\Sigma w_i y_i^2$, with the same numerator, where b_i is the fitted background. It is background-dependent and supplements the conventional value.

$$Rwp = 100 \sqrt{\frac{S}{\Sigma w_i {y_i}^2}}$$

Table S3. Full-signal and net-signal residuals.

State	Rwp (%)	Net-profile Rwp (%)	Σb/Σy (%)
As-spun	2.055517	17.174787	92.6818
After DSC	3.541954	18.715449	89.3952

Figure S1. Net diffraction profiles in the 19.5–46° interval. Observed minus background and calculated minus background are normalized by the maximum observed net intensity of each state. Difference curves are offset by −0.2. The actual residuals remain visible.

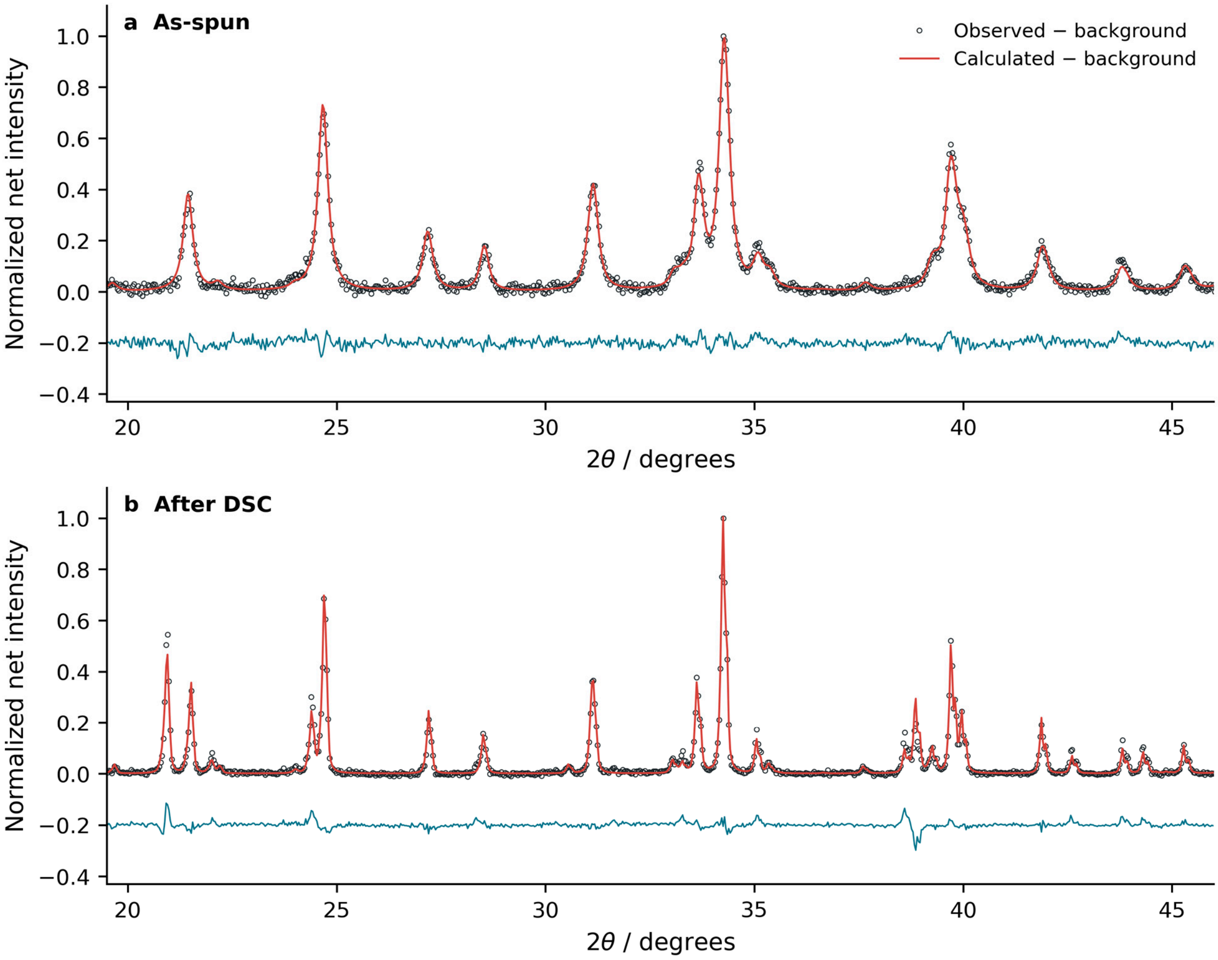

S4 Local geometry and crystallographic reuse

The after-DSC $CaAuMg_2$ model gives two Au–Mg contacts of 2.777(12) Å and four of 2.826(7) Å. Four nearest Mg contacts are A = 3.452(23) Å, B = 3.084(17) Å twice and C = 3.282(23) Å. Uncertainties propagate the refined covariance, including lattice and positional correlations, and remain conditional on texture/profile assumptions. Full values are in data/contact_uncertainties.json.

The comparison with Kersting et al. uses their $CaAuMg_2$ powder cell and the $SrPdMg_2$ fractional coordinates underlying the transferred-coordinate geometry. It is not a comparison with an independent single-crystal refinement of $CaAuMg_2$. The reconstructed A, B, and C contacts are approximately 3.674, 2.999, and 3.147 Å. The primary reference is [10] in the revised manuscript.

Five CIF files describe known compounds in the two states: $CaAuMg_2$ and Mg_2Ca before and after treatment, and Mg_3Au after treatment. They contain explicit space-group operations, multiplicities, cell uncertainties, and refinement notes. For the as-spun ternary, transferred positions have no independently measured positional uncertainty. Z is 4 for $CaAuMg_2$ and Mg_2Ca, and 6 for Mg_3Au.

data/CIF_validation.json records successful local parsing and consistency of chemical formula × Z with occupied site multiplicities. This is a local consistency check, not an IUCr checkCIF report or COD acceptance. The files can be used for structural visualization and calculation and prepared for public deposition after bibliographic completion and repository validation. No new-compound discovery or deposition identifier is claimed.

S5 DSC export

The heating branch contains 24,038 recorded points and reaches 399.795 °C. It is plotted without smoothing or baseline subtraction. The cooling branch contains 6,014 points, of which 5,971 report exactly −11.179414 mW, and is not used to infer transformation temperatures. Argon is the author-confirmed atmosphere. No glass-transition, crystallization, or melting temperature, nor transition enthalpy, is inferred from the smooth broad heating curvature.

S6 Reproduction

Open either final .gpx project in GSAS-II release 5799 to inspect embedded data, structural models, controls, and calculated profiles. The .lst files retain native refinement results. code/recalculate_projects.py optionally reruns copies in a separate output directory using an existing GSAS-II 5799 installation; it does not overwrite the delivered projects.

Run python code/make_figures.py to regenerate the SEM crop and experimental plots from the included numerical files. Run python code/render_structures.py to regenerate coordinate-based atom renderings. These plotting scripts require NumPy, Matplotlib, and Pillow. The structural renderings use the crystallographic coordinates.

S7 Positional refinement and correlated residuals

The comparison concerns the archived fixed-coordinate and four-coordinate refinements of the after-DSC pattern. Both use the same 3142 data points, wavelengths, point weights, background order, and profile treatment. The fixed-coordinate baseline has y(Mg) = 0.3593039, z(Mg) = 0.0579114, y(Au) = 0.7704073 and y(Ca) = 0.0532013 (Table S2); it is not the transferred $SrPdMg_2$ coordinate set. The variable lists differ by exactly the four independent positional parameters. The nested-model calculation uses the archived GSAS-II least-squares sums and parameter counts [S1]. No additional fit is performed.

Table S4. Archived fit statistics and diagnostics of the weighted profile residuals.

Quantity	Fixed coordinates	Refined coordinates
Number of observations	3142	3142
Independent parameters	48	52
Weighted least-squares sum S	10910.9565	10274.7155
Rwp / %	3.649971	3.541954
Durbin–Watson d	0.906	0.946
Lag-one residual correlation	0.547	0.527

For a fixed-coordinate model with p_0 parameters and an expanded model with p_1 parameters, the nominal statistic is [S1]:

$$F = \frac{\frac{S_0 - S_1}{p_1 - p_0}}{\frac{S_1}{N - p_1}} = 47.84$$

Here $p_1 - p_0 = 4$ and $N - p_1 = 3090$. The weighted least-squares sum decreases by 5.831%. Assuming independent, appropriately weighted errors and a locally linear model, the upper-tail probability is approximately 4.42×10^{-39}. This nominal probability is conditional on assumptions that the measured residuals do not satisfy adequately; it is not reported as a calibrated experimental significance level.

Serial correlation was evaluated from $e_i = \sqrt{w_i}(y_i - y_{calc,i})$, using the Durbin–Watson diagnostic [S2]:

$$d = \frac{\Sigma_{i=2}{}^{N}(e_i - e_{i-1})^2}{\Sigma_{i=1}{}^{N} e_i{}^2} = 0.946\, for\ the\ refined\ model$$

The corresponding Pearson correlation of adjacent weighted residuals is 0.527. Positive serial correlation and remaining systematic profile mismatch make an independent-point interpretation inappropriate. The largest absolute correlation between an individual positional parameter and any other fitted parameter in the local covariance matrix is 0.411, between y(Mg) and the ternary C(6,4) texture coefficient. This local matrix does not capture the systematic effect of changing the texture model. The SH4/SH6 shift in z(Mg) and the alternative-start test in Table S2 therefore remain essential to interpretation. The nominal F test supports improvement over the specified baseline under its assumptions; it does not establish a unique local geometry or a new structure type.

The calculation is reproduced by code/diagnostics.py. The numerical output is data/nested_model_diagnostic.json; the fixed-coordinate statistics and variable list are retained in data/sensitivity_tests.json. The diagnostic uses archived statistics for the F ratio and the corresponding exported profiles for residual correlation.

S8 Reflection indexing and peak overlap

The identifiers of the structural sources are Mg_3Au: COD 1510235, Daams and van Vucht [15]; Mg_2Ca: COD 4313238, Amerioun et al. [16]; $CaAuMg_2$: the powder cell and structural assignment in Kersting et al. [10], together with the $MgCuAl_2$-type site arrangement [17]. These are the sources used to calculate the reference patterns. No verified ICDD PDF/JCPDS card numbers were used or assigned.

The complete file data/indexed_reflections.csv contains 782 calculated reflections within the measured angular ranges of the two final models. Each entry gives the phase, source, hkl, d spacing, ideal Bragg $K\alpha_1$ position, specimen-displacement-corrected $K\alpha_1/K\alpha_2$ positions, calculated squared structure factor, preferred-orientation correction, and fitted component FWHM. The CSV is a model index, not a list of 782 separately resolved experimental peaks. Its two angle conventions are distinguished because specimen displacement shifts the fitted peak center from the ideal Bragg position calculated from d alone.

Table S5. Selected after-DSC reflections. The listed 2θ values are calculated Kα$_1$ positions on the experimental angular scale, including the fitted specimen displacement. Δother is the smallest separation to a calculated reflection of another included phase, considering both Kα components without an intensity cutoff. Component FWHM values describe the adopted profile, not an independent instrumental calibration.

Phase	(hkl)	d / Å	2θ / °	FWHM / °	Δother / °
Mg_3Au	(002)	4.2416	20.950	0.051	0.573
Mg_3Au	(110)	4.0382	22.016	0.051	0.197
Mg_3Au	(111)	3.6462	24.416	0.053	0.303
$CaAuMg_2$	(111)	3.6021	24.719	0.051	0.109
$CaAuMg_2$	(022)	3.2764	27.218	0.052	1.308
Mg_2Ca	(110)	3.1290	28.526	0.059	1.239
Mg_2Ca	(112)	2.6636	33.642	0.063	0.262
Mg_2Ca	(201)	2.6181	34.244	0.064	0.027
$CaAuMg_2$	(131)	2.6161	34.271	0.057	0.027
Mg_3Au	(300)	2.3315	38.607	0.063	0.575
Mg_3Au	(113)	2.3163	38.870	0.063	0.312
$CaAuMg_2$	(113)	2.2684	39.724	0.061	0.441

Mg_3Au (002), $CaAuMg_2$ (022) and Mg_2Ca (110) offer comparatively separated checks within the adopted phase set. In contrast, the Mg_2Ca (201)/$CaAuMg_2$ (131) pair is separated by only 0.027°, smaller than either fitted component width. This overlapped feature cannot independently identify either phase. The combination of indexed positions, less-overlapped regions, and full-profile fits supports the assignments, while broad as-spun peaks, texture-dependent intensities, and potential unmodeled minor phases limit completeness and quantitative interpretation.

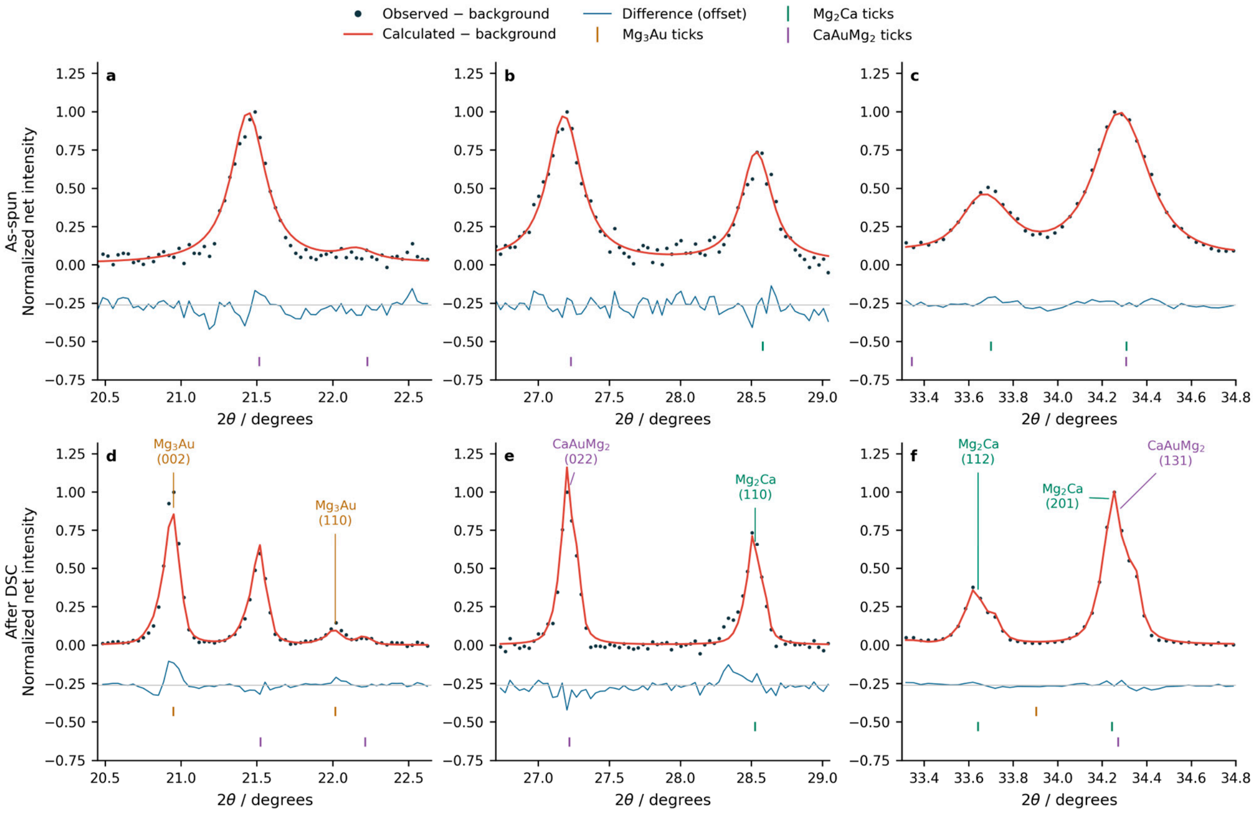

Figure S2. Enlarged regions of the unchanged measured and calculated profiles for (a–c) the as-spun ribbon and (d–f) the specimen after DSC. Observed and calculated backgrounds are subtracted using the fitted background. Each panel is normalized by its own maximum observed net intensity; the difference is offset by −0.26. Colored tick rows show calculated Kα_1 positions for Mg_3Au, Mg_2Ca and $CaAuMg_2$, from top to bottom; Kα_2 is included in the calculated profile. Selected associated planes are labeled in the after-DSC panels. The as-spun model contains no Mg_3Au, so that tick row is empty. Panels (b,e) illustrate separated $CaAuMg_2$/Mg_2Ca checks; panels (c,f) include the strong Mg_2Ca (201)/$CaAuMg_2$ (131) overlap. These plots assess the adopted models and do not constitute an independent diffraction experiment.

The plotted profiles are reproduced by code/plot_diagnostics.py. The reflection index and widths are extracted from the existing GSAS-II models, with the Cu Kα_2 positions calculated using the stated wavelengths and specimen displacement. The auxiliary statistics and indexing do not change the selected phase fractions, atomic coordinates, or final project files.

Supplementary references

[S1] Hamilton, W.C. Significance tests on the crystallographic R factor. Acta Crystallogr. 1965, 18, 502–510. https://doi.org/10.1107/S0365110X65001081.

[S2] Hill, R.J.; Flack, H.D. The use of the Durbin–Watson d statistic in Rietveld analysis. J. Appl. Crystallogr. 1987, 20, 356–361. https://doi.org/10.1107/S0021889887086485.
